# Supplementary material for: Mathematical and computational modeling for organic and insect frass fertilizer production: A systematic review
Source: PLoS One. 2025 Jan 24;20(1):e0292418. doi: 10.1371/journal.pone.0292418 (PMC11760587; doi:10.1371/journal.pone.0292418)
Supplement: Attachment — Submitted filename: Response to Reviewers.pdf [file pone.0292418.s002.pdf]

To The Editor,

PLOS ONE.

10th September 2024.

Dear Sir/Madam,

RE: <u>RESPONSE TO REVIEWER'S COMMENTS: MANUSCRIPT NO.: PONE-D-23-28302</u>

Many thanks to you for your helpful feedback. We have tried to address all your concerns to the best of our abilities. Kindly help us with your further concerns (if any) to improvise our paper. Below we are providing our responses to the comments and actions that we took to address them. The responses are highlighted in blue.

Here is the updated funding information for you to kindly upload it in the system:

=======================

The authors gratefully acknowledge the financial support for this research by the following organizations and agencies: Australian Centre for International Agricultural Research (ACIAR) (ProteinAfrica –Grant No: LS/2020/154), Global Affairs Canada (BRAINS project: P011585), Novo Nordisk Foundation (RefIPro: NNF22SA0078466), the Rockefeller Foundation (WAVE-IN—Grant No: 2021 FOD 030); Bill and Melinda Gates Foundation (INV-032416); IKEA Foundation (G-2204-02144), European Commission (HORIZON 101060762 NESTLER and HORIZON 101136739 INNOECOFOOD), the Curt Bergfors Foundation Food Planet Prize Award; Norwegian Agency for Development Cooperation, the Section for Research, Innovation, and Higher Education grant number RAF–3058 KEN–18/0005 (CAP–Africa); the Swedish International Development Cooperation Agency (Sida); the Swiss Agency for Development and Cooperation (SDC); the Australian Centre for International Agricultural Research (ACIAR); the Norwegian Agency for Development Cooperation (Norad); the German Federal Ministry for Economic Cooperation and Development (BMZ); the Federal Democratic Republic of Ethiopia; and the Government of the Republic of Kenya. The funders had no role in study design, data collection and analysis, decision to publish, or

preparation of the manuscript. The views expressed herein do not necessarily reflect the official opinion of the donors.

=======================

Yours faithfully,

Dr. Kennedy Senagi

Email: ksenagi@icipe.org

BELOW IS OUR RESPONSE TO THE REVIEWER'S COMMENTS IN BLUE

===

RESPONSE 2

===

PONE-D-23-28302R1

Mathematical and Computational Modeling for Organic and Insect Frass Fertilizer Production: A Systematic Review

Dear Dr. Senagi,

We've checked your submission and before we can proceed, we need you to address the following issues:

1. We note that your manuscript is not formatted using one of PLOS ONE's accepted file types. Please reattach your manuscript as one of the following file types: .doc, .docx, .rtf, or .tex (accompanied by a .pdf).

If your submission was prepared in LaTex, please submit your manuscript file in PDF format and attach your .tex file as "other."

- ⇨ We have uploaded the .tex files and other documents. Thanks

2. Thank you for updating your data availability statement. You note that your data are available within the Supporting Information files, but no such files have been included with your submission. At this time we ask that you please upload your minimal data set as a Supporting Information file, or to a public repository such as Figshare or Dryad. Please also ensure that when you upload your file you include separate captions for your supplementary files at the end of your manuscript.

As soon as you confirm the location of the data underlying your findings, we will be able to proceed with the review of your submission.

- ⇨ Apologies, we have edited the Data Availability statement to "No – some restriction will apply".
- ⇨ We have left this note to describe where the data may be found, "Data resources used to write this systematic review article are well referenced within the manuscript".
- ⇨ Ideally, this is a Systematic Review, and we have no data available. However, all analysed articles are well referenced in the article.

3. Please note that funding information should not appear in any section or other areas of your manuscript. We will only publish funding information present in the Funding Statement section of the online submission form. Please remove any funding-related text from the manuscript.

- ⇨ We have removed all funding related information from the manuscript. Thank you.

==

RESPONSE 1

==

Authors of PONE-D-23-28302

PONE-D-23-28302

Mathematical and Computational Modeling in Organic Fertilizer Production: A Systematic Review

PLOS ONE

Dear Dr. Senagi,

Thank you for submitting your manuscript to PLOS ONE. After careful consideration, we feel that it has merit but does not fully meet PLOS ONE's publication criteria as it currently stands. Therefore, we invite you to submit a revised version of the manuscript that addresses the points raised during the review process.

Please see the comments from three reviewers below.

Please submit your revised manuscript by Sep 01 2024 11:59PM. If you will need more time than this to complete your revisions, please reply to this message or contact the journal office at plosone@plos.org. When you're ready to submit your revision, log on to https://www.editorialmanager.com/pone/ and select the 'Submissions Needing Revision' folder to locate your manuscript file.

Please include the following items when submitting your revised manuscript:

- A rebuttal letter that responds to each point raised by the academic editor and reviewer(s). You should upload this letter as a separate file labeled 'Response to Reviewers'.

  ⇨ That's included

- A marked-up copy of your manuscript that highlights changes made to the original version. You should upload this as a separate file labeled 'Revised Manuscript with Track Changes'.

  ⇨ That's included

- An unmarked version of your revised paper without tracked changes.
  You should upload this as a separate file labelled 'Manuscript'.

  ⇨ That's included

If you would like to make changes to your financial disclosure, please include your updated statement in your cover letter. Guidelines for resubmitting your figure files are available below the reviewer comments at the end of this letter.

If applicable, we recommend that you deposit your laboratory protocols in protocols.io to enhance the reproducibility of your results. Protocols.io assigns your protocol its own identifier (DOI) so that it can be cited independently in the future. For instructions see: https://journals.plos.org/plosone/s/submission-guidelines#loc-laboratory-protocols. Additionally, PLOS ONE offers an option for publishing peer-reviewed Lab Protocol articles, which describe protocols hosted on protocols.io. Read more information on sharing protocols at https://plos.org/protocols?utm_medium=editorial-email&utm_source=authorletters&utm_campaign=protocols.

We look forward to receiving your revised manuscript.

Kind regards,

Hanna Landenmark

Staff Editor

PLOS ONE

Journal Requirements:

When submitting your revision, we need you to address these additional requirements.

1. Journal requirements:

When submitting your revision, we need you to address these additional requirements.

1. Please ensure that your manuscript meets PLOS ONE's style requirements, including those for file naming. The PLOS ONE style templates can be found at https://journals.plos.org/plosone/s/file?id=wjVg/PLOSOne_formatting_sample_main_body.pdf and https://journals.plos.org/plosone/s/file?id=ba62/PLOSOne_formatting_sample_title_authors_affiliations.pdf

   ⇨ That is corrected. We have now followed the PLOS ONE template available at https://journals.plos.org/plosone/s/latex .

2. We note that the grant information you provided in the 'Funding Information' and 'Financial Disclosure' sections do not match.

When you resubmit, please ensure that you provide the correct grant numbers for the awards you received for your study in the 'Funding Information' section.

   ⇨ In the revised articles, we have included the updated "Funding Information" in the Acknowledgement Section. This (updated) information is also appended on the cover letter (in Page 1). We have also provided the grant numbers. However, note that, the institution has core donors that are not issued with grant numbers.

3. Thank you for stating the following in the Acknowledgments Section of your manuscript: "The authors gratefully acknowledge the financial support for this research by the following organizations and agencies: the Swedish International Development Cooperation Agency (Sida); the Swiss Agency for Development and

Cooperation (SDC); the Australian Centre for International Agricultural Research (ACIAR); the Federal Democratic Republic of Ethiopia; and the Government of the Republic of Kenya. The views expressed herein do not necessarily reflect the official opinion of the donors."

- ⇨ The content of the Acknowledgement Section is updated. It is appended on the cover letter (in Page 1).

We note that you have provided funding information that is currently declared in your Funding Statement. However, funding information should not appear in the Acknowledgments section or other areas of your manuscript. We will only publish funding information present in the Funding Statement section of the online submission form.

Please remove any funding-related text from the manuscript and let us know how you would like to update your Funding Statement. Currently, your Funding Statement reads as follows: "The authors gratefully acknowledge the financial support for this research by the following organizations and agencies: the specific restricted project donor (written out in full) and grant number; the Swedish International Development Cooperation Agency (Sida); the Swiss Agency for Development and Cooperation (SDC); the Australian Centre for International Agricultural Research (ACIAR); the Norwegian Agency for Development Cooperation (Norad); the Federal Democratic Republic of Ethiopia; and the Government of the Republic of Kenya. The views expressed herein do not necessarily reflect the official opinion of the donors."

Please include your amended statements within your cover letter; we will change the online submission form on your behalf.

- ⇨ We have updated the Acknowledgement with an updated funding statement. We have also appended the same on the cover letter (in Page 1) for you to kindly update it in the system. Thank you.

4. We note that your Data Availability Statement is currently as follows: [All relevant data are within the manuscript and its Supporting information files]

   Please confirm at this time whether or not your submission contains all raw data required to replicate the results of your study. Authors must share the "minimal data set" for their submission. PLOS defines the minimal data set to consist of the data required to replicate all study findings reported in the article, as well as related metadata and methods (https://journals.plos.org/plosone/s/data-availability#loc-minimal-data-set-definition).

   For example, authors should submit the following data:

   - The values behind the means, standard deviations and other measures reported;
   - The values used to build graphs;
   - The points extracted from images for analysis.

   Authors do not need to submit their entire data set if only a portion of the data was used in the reported study.

   If your submission does not contain these data, please either upload them as Supporting Information files or deposit them to a stable, public repository and provide us with the relevant URLs, DOIs, or accession numbers. For a list of recommended repositories, please see https://journals.plos.org/plosone/s/recommended-repositories.

   If there are ethical or legal restrictions on sharing a de-identified data set, please explain them in detail (e.g., data contain potentially sensitive information, data are owned by a third-party organization, etc.) and who has imposed them (e.g., an ethics committee). Please also provide contact information for a data access committee, ethics committee, or other institutional body to which data requests may be sent. If data are owned by a third party, please indicate how others may request data access

   ⇨ This study collected secondary data (i.e., scientific articles and other online resource). All these recourses are well references in the article.

5. Please review your reference list to ensure that it is complete and correct. If you have cited papers that have been retracted, please include the rationale for doing so in the manuscript text, or remove these references and replace them with relevant current references. Any changes to the reference list should be mentioned in the rebuttal letter that accompanies your revised manuscript. If you need to cite a retracted article, indicate the article's retracted status in the References list and also include a citation and full reference for the retraction notice.
   - ⇨ We have reviewed all the references in this article.

[Note: HTML markup is below. Please do not edit.]

Reviewers' comments:

Reviewer's Responses to Questions

Comments to the Author

1. Is the manuscript technically sound, and do the data support the conclusions?

The manuscript must describe a technically sound piece of scientific research with data that supports the conclusions. Experiments must have been conducted rigorously, with appropriate controls, replication, and sample sizes. The conclusions must be drawn appropriately based on the data presented.

Reviewer #1: Yes

Reviewer #2: Yes

Reviewer #3: Yes

---

2. Has the statistical analysis been performed appropriately and rigorously?

Reviewer #1: Yes

Reviewer #2: N/A

Reviewer #3: Yes

---

3. Have the authors made all data underlying the findings in their manuscript fully available?

   The PLOS Data policy requires authors to make all data underlying the findings described in their manuscript fully available without restriction, with rare exception (please refer to the Data Availability Statement in the manuscript PDF file). The data should be provided as part of the manuscript or its supporting information, or deposited to a public repository. For example, in addition to summary statistics, the data points behind means, medians and variance measures should be available. If there are restrictions on publicly sharing data—e.g. participant privacy or use of data from a third party—those must be specified.

   Reviewer #1: Yes

   Reviewer #2: Yes

   Reviewer #3: Yes

---

4. Is the manuscript presented in an intelligible fashion and written in standard English?

   PLOS ONE does not copyedit accepted manuscripts, so the language in submitted articles must be clear, correct, and unambiguous. Any typographical or grammatical errors should be corrected at revision, so please note any specific errors here.

   Reviewer #1: Yes

   Reviewer #2: Yes

   Reviewer #3: Yes

5. Review Comments to the Author

Please use the space provided to explain your answers to the questions above. You may also include additional comments for the author, including concerns about dual publication, research ethics, or publication ethics. (Please upload your review as an attachment if it exceeds 20,000 characters)

Reviewer #1:

Dear authors,

I would like to extend my sincere congratulations to the authors for their exceptional work on this systematic review. The article well-written, and presenting the valuable from previous studies. The efforts in summarizing and critically evaluating the included studies have provided readers with valuable insights into the current state of knowledge in this field.

I have a few comments to be amended from your manuscript:

1) Page: 4, Table 2:

What is the meaning of symbol "*"?

Maybe the author can mention as foot note below the table regarding the meaning of "*".

⇨ This has been addressed. Please check the caption of Table 2, Page 5.

2)The references format inconsistent. Kindly check the format of manuscript provided by PLOS ONE.

⇨ We have used the standard PLOS ONE manuscript template and adopted the same referencing style.

I hope that your findings will guide future research efforts. Once again, congratulations on your outstanding systematic review.

⇨ Thank you!

Reviewer #2:

Thank you so much for doing this attractive systematic review.

It has been written according to the PRISMA writing standard. However, once again, please check the manuscript in terms of heading and subheading with the PRISMA checklist.

⇨ Heading and subheading issues have been checked accordingly.

In the discussion section, I am not sure whether the key findings is necessary or not. I think it is better to adhere to the journal`s instruction for authors and omit it if possible. Conclusion is enough.

⇨ We have removed the "Key Finding" section and a summary of it integrated in the Conclusion section. Please check Line 704-713.

⇨ Thank you!

Reviewer #3:

As the statistical reviewer I will focus on methods and reporting. Overall, this is a well conducted and reported study and I only have a few points to raise.

Major

1) it was not clear if the authors searched the reference lists of the identified papers, for any relevant papers that were not returned by their searches. it is customary to do that and

also report on the numbers and percentages missed. A low percentage is an indication of a good / working well search strategy.

- ⇨ Thank you for the comment.
- ⇨ We revised the search key query and words across as web of science, Scopus, and Google Scholar. On the internet, it's not easy identify scientific resources that were left out. Ideally, the query was structured with keywords (Shown in Table 3) to do a thorough search just like other systematic reviews have done (such as https://doi.org/10.3390/medicina57060538, https://doi.org/10.1109/ACCESS.2020.2973006, https://doi.org/10.1155/2021/6619364). It is from that search that we used the inclusion and exclusion criteria (in Table 4) to identify the relevant sources for this systematic review and leave out what was not relevant as shown in Figure 1. Nonetheless, we further modified the search query to a detailed search as shown in Table 3; perhaps to gather any missing literature. We identified recent developments of insect frass as an organic fertilizer. That is, in 2021, the European Union accepted insect frass as organic fertilizer. We have updated this article with this content. Please check:
    - Abstract (2nd sentence)
    - Line 58 – 72
    - Line 118
    - Highlighted text in Table 2 and 3
    - Line 186, 188, 191, 194,
    - Figure 3
    - Highlighted items (33, 34, and 35) in Table 5
    - Highlighted items in Table 6
    - Line 219-243
    - Line 653-655
    - Line 747
    - Line 768

2) I have concerns about the risk of bias tool used, it is not clear which one it is. is it ROB-2? this tool is specifically for RCTs and it is not clear how the domains map and are relevant to the studies included in this review. I'm not even sure an appropriate risk of bias tool exists, since there is no comparison really. Anyway, the authors need to reflect and explain in the next iteration of the paper.

- ⇨ Indeed, ROB is widely used in randomized controlled trials (RCTs) in the medical field. However, we note that the ROB can be customized/tailored to assess risk of specific items (in this case: assumptions, variables, formulation, resolution/validation, and simulation) in a literature study. Nonetheless, other articles out of the medical field that have utilized ROB are:
  - https://doi.org/10.1136/bmj.n2281
  - https://doi.org/10.7326/M18-1376
  - https://doi.org/10.1016/j.jid.2016.08.021

Minor

1) Meta analysis is mentioned a couple of times in the paper, I suggest not mentioning it since it is irrelevant (nothing to pool).

- ⇨ This has been corrected, Thank you!

PLOS authors have the option to publish the peer review history of their article (what does this mean?). If published, this will include your full peer review and any attached files.

If you choose "no", your identity will remain anonymous but your review may still be made public.

Do you want your identity to be public for this peer review? For information about this choice, including consent withdrawal, please see our Privacy Policy.

Reviewer #1: No

Reviewer #2: **Yes:** Mahin Nomali

Reviewer #3: No

[NOTE: If reviewer comments were submitted as an attachment file, they will be attached to this email and accessible via the submission site. Please log into your account, locate the manuscript record, and check for the action link "View Attachments". If this link does not appear, there are no attachment files.]

While revising your submission, please upload your figure files to the Preflight Analysis and Conversion Engine (PACE) digital diagnostic tool, https://pacev2.apexcovantage.com/. PACE helps ensure that figures meet PLOS requirements. To use PACE, you must first register as a user. Registration is free. Then, login and navigate to the UPLOAD tab, where you will find detailed instructions on how to use the tool. If you encounter any issues or have any questions when using PACE, please email PLOS at figures@plos.org. Please note that Supporting Information files do not need this step.

⇨ This has been considered during revisions, Thank you!
